# Thermally Active Medium-Density Fiberboard (MDF) with the Addition of Phase Change Materials for Furniture and Interior Design

**DOI:** 10.3390/ma17164001

**Published:** 2024-08-12

**Authors:** Julia Dasiewicz, Anita Wronka, Aleksandra Jeżo, Grzegorz Kowaluk

**Affiliations:** 1 Faculty of Wood Technology, Warsaw University of Life Sciences-SGGW, Nowoursynowska St. 159, 02-787 Warsaw, Poland; s209150@sggw.edu.pl; 2 Institute of Wood Science and Furniture, Warsaw University of Life Sciences—SGGW, Nowoursynowska St. 159, 02-776 Warsaw, Poland; aleksandra_jezo@sggw.edu.pl

**Keywords:** medium-density fiberboard, MDF, wood modification, thermal conductivity, PCMs

## Abstract

No matter where we reside, the issue of greenhouse gas emissions impacts us all. Their influence has a disastrous effect on the earth’s climate, producing global warming and many other irreversible environmental impacts, even though it is occasionally invisible to the independent eye. Phase change materials (PCMs) can store and release heat when it is abundant during the day (e.g., from solar radiation), for use at night, or on chilly days when buildings need to be heated. As a consequence, buildings use less energy to heat and cool, which lowers greenhouse gas emissions. Consequently, research on thermally active medium-density fiberboard (MDF) with PCMs is presented in this work. MDF is useful for interior design and furniture manufacturing. The boards were created using pine (*Pinus sylvestris* L.) and spruce (*Picea abies* L.) fibers, urea–formaldehyde resin, and PCM powder, with a phase transition temperature of 22 °C, a density of 785 kg m^−3^, a latent heat capacity of 160 kJ kg^−1^, a volumetric heat capacity of 126 MJ m^−3^, a specific heat capacity of 2.2 kJ kgK^−1^, a thermal conductivity of 0.18 W mK^−1^, and a maximum operating temperature of 200 °C. Before resination, the wood fibers were divided into two outer layers (16%) and an interior layer (68% by weight). Throughout the resination process, the PCM particles were solely integrated into the inner layer fibers. The mats were created by hand. A hydraulic press (AKE, Mariannelund, Sweden) was used to press the boards, and its operating parameters were 180 °C, 20 s/mm of nominal thickness, and 2.5 MPa for the maximum unit pressing pressure. Five variants of MDF with a PCM additive were developed: 0%, 5%, 10%, 30%, and 50%. According to the study, scores at the MOR, MOE, IB, and screw withdrawal resistance (SWR) tests decreased when PCM content was added, for example, MOE from 3176 to 1057 N mm^−2^, MOR from 41.2 to 11.5 N mm^−2^, and IB from 0.78 to 0.27 N mm^−2^. However, the results of the thickness swelling and water absorption tests indicate that the PCM particles do not exhibit a substantial capacity to absorb water, retaining the dimensional stability of the MDF boards. The thickness swelling positively decreased with the PCM content increase from 15.1 to 7.38% after 24 h of soaking. The panel’s thermal characteristics improved with the increasing PCM concentration, according to the data. The density profiles of all the variations under consideration had a somewhat U-shaped appearance; however, the version with a 50% PCM content had a flatter form and no obvious layer compaction on the panel surface. Therefore, certain mechanical and physical characteristics of the manufactured panels can be enhanced by a well-chosen PCM addition.

## 1. Introduction 

Growing energy consumption and greenhouse gas emissions are driving developments in renewable energy sources and energy efficiency [1,2,3,4]. They also pose a significant barrier to global development [5,6], as energy is essential to the expansion of the automobile industry as well as societal advancement. Concern over the rising energy use and its negative effects on the environment, human health, and climate change is developing on a global scale. Currently, 40% of the world’s energy is consumed by the building industry; by 2050, this percentage is predicted to climb to 50% [7]. The primary uses of energy are cooking, lighting, entertainment, different types of sanitation and cleaning, and space heating and cooling. Buildings employ a variety of energy sources, such as pipeline gas, electricity, gasoline, coal, natural gas, liquefied petroleum gas (LPG), solar energy, biogas, and biomass [8,9]. Buildings throughout the world now require lower energy usage. A significant portion of energy use may be cut by switching to clean and renewable energy systems for lighting, cooling, and heating. It is not always feasible to rely entirely on renewable energy sources such as wind and solar energy [10,11,12].

The need for more reasonably priced, high-capacity thermal energy storage systems has arisen as a result. Phase change materials (PCMs), or phase change material, is one of numerous energy storage and control technologies [13]. It is acknowledged that employing phase change materials can enhance a building’s energy management. PCMs have the ability to store and release thermal energy in the form of latent heat due to their high energy storage density and isothermal phase transition [14,15]. This explains why they are often used in thermal energy storage systems, such as air conditioning in modern buildings [16], thermal management in electronic equipment [17], and smart textiles [18]. However, the leaking of liquid during melting is a major problem that hinders the practical application of both organic solid and liquid PCMs [19,20]. A material is said to be latent because its thermal energy is held between its molecules until it transitions from one phase to another. Matter is made up of molecules joined by chemical bonds. These chemical connections allow heat to be both emitted and retained. Heat must be stored (absorbed) for the PCM to function. This dissolves the bonds holding the material together and turns them into a liquid [21]. Latent heat is the amount of energy needed to change a material’s physical condition at a given temperature [22]. This can boost a building’s thermal mass and raise its energy efficiency. Stable PCM composites may be created by directly integrating, dipping, encapsulating, shape-stabilizing, and combining PCMs with other materials and structural components [23,24,25,26,27]. Numerous studies have previously been conducted in this field, and new methods are always being created. For instance, Kumar et al. tested combining solar energy and the HS22 PCM to heat space in frigid climates, keeping a room temperature of 10 to 20 °C even while the outside temperature was -10 to 0 ^o^C [28]. Furthermore, according to a study by Guimarães et al., adding PCMs to building materials improves energy efficiency by lowering room temperature swings [29]. Wieland emphasized using finned heat exchangers to accelerate heat transfer rates and PCMs like paraffin wax in storing thermal energy [30]. To reduce energy costs and boost hot water output when combined with electric heaters, Djeffal et al. created a PCM blend of animal fat and paraffin wax for hot water systems [31]. According to Mohseni et al., PCMs have even been used in phase change memory technology [32], and their potential for data storage applications is demonstrated by the study. Moreover, PCMs can lessen mechanical stresses in concrete components by absorbing heat during the curing process, as demonstrated by Fabiani et al. [33]. 

The use of phase change materials (PCMs) significantly enhances the thermal characteristics of wood-based panels. According to studies, PCM-impregnated wood panels offer better qualities, including resistance to biological assaults, thermal stability, and effective heat storage and release during phase shift processes [34,35]. The creation of particleboard with modified cassava starch as a binder and Ironwood red chips as a raw material has shown enhanced mechanical qualities appropriate for interior panels and ceilings, underscoring the adaptability and promise of PCMs in the production of wood-based panels [36]. One commonly used method of applying PCMs in combination with wood is its impregnation. Using this method, researchers have prepared a flooring panel that not only exhibits improved thermal properties but also shows increased scratch resistance, especially in the solid phase of the PCM. In the liquid phase, the hardness is not lower than that of wood, which is a positive aspect. The flammability of the impregnated elements was also investigated, and it was unequivocally stated that using such structural elements without additional protection can be dangerous, as PCMs increase the flammability of wood elements after impregnation [37]. Yang and colleagues were able to develop a novel phase transition material with a strong reversible thermochromic capacity by including a thermochromic chemical onto delignified wood (DW) slides [38]. Comparably, Ma et al. [39] used the caprylic–palmitic acid treatment on DW slides to create a stable phase change composite (PCC) with a resting phase transition temperature of 23.4 °C. According to both investigations, the removal of lignin enhanced PCM adsorption by opening the catheter chambers and DW slides and increasing their permeability. In the study by [40], delignification and impregnation with myristyl alcohol (MA) were used to create stable phase change composites (PCCs) based on wood powder (WF), a by-product of the wood industry. Phase change composites, urea–formaldehyde (UF) resin, and delignified wood flour (dWF)/myristyl alcohol impregnation have been used to generate composite panels. This approach provides a practical route to widespread use in energy-related domains. This straightforward and flexible method offers significant potential for the large-scale manufacturing of shape-stable phase change composites and may be expanded to further temperature control applications. In research by Jeong et al. [41], the authors looked at the use of microencapsulated phase change material (MPCM) in wood-based flooring to introduce MPCM into construction materials. The incorporation of hemp for interior wall panels with melamine and formaldehyde as the outside coating and a microencapsulated phase change material (PCM) as the core material was also assessed [42]. The test employed organic PCM-S28, which has a melting point between 25 and 29 °C. To create the test panels, 10% Kleiberit urea-based formaldehyde resin (UF) was employed as a binder during the cold pressing procedure. The test panels were 25 mm thick and had a density of 310 ± 20 kg m^−3^, which satisfied the specifications for low-density panels. When 5% nanocapsules were inserted during the panel production process, the heat capacity improved by 28%. Another example of PCM application in wood is multifunctional wood-based phase change composite materials with magnetic additions created by impregnating purified balsa wood with a combination of 1-tetradecanol and Fe_3_O_4_ nanoparticles. The results revealed that these composites had a large energy storage capacity, outstanding thermal reliability even after 100 heating and cooling cycles, and good thermal stability up to 112 °C while remaining shape stable. Furthermore, the incorporation of Fe_3_O_4_ nanoparticles enhances the composites’ magnetic characteristics and increases the efficiency of solar-to-thermal energy conversion. The materials also display a magnetothermal effect, which is the increase in temperature caused by a changing magnetic field [28,29,30,31,32,33,34,35,36,37,38,39,43,44].

Due to this investigation, scanning electron microscopy (SEM, FESEM, Ziess, Germany) examination verified that MPCM was evenly distributed throughout the adhesive [40,41]. The features of a thermal energy storage material were established for this composite by differential scanning calorimetry (DSC) investigation.

When it comes to thermal energy storage applications, organic phase change materials (PCMs) are quite advantageous [42,43]. They are appropriate for a range of temperature control applications because of their high latent heat storage capacity and wide range of phase transformation temperatures [4]. The synthesis of fatty acid amides (FAAms) with high purity and efficiency is one example of how the usage of bio-based organic PCMs from sources like soybean oil may support environmental sustainability by providing a green alternative to petroleum-based PCMs [1]. Additionally, the ability to design eutectic PCMs by combining different organic PCMs allows for the optimization of thermal conductivity by adding materials, thereby addressing poor thermal conductivity and increasing their applicability in a variety of real-world scenarios. Thermal properties like phase transition temperature and enthalpy of fusion can be adjusted. Because of these benefits, organic PCMs are attractive options for effective and sustainable thermal management in a number of domains, such as the use of solar energy, energy-efficient buildings, and electronic device thermal management [4].

When taken together, these studies show how versatile and successful PCM is in a range of applications, including building energy systems, thermal energy storage, and space heating. Therefore, this research aims to create a thermally active medium-density fiberboard (MDF) with PCM powder. MDF can be useful for interior design and furniture manufacturing.

## 2. Materials and Methods

### 2.1. Materials

In the present study, medium-density fiberboard (MDF) was produced under laboratory conditions from pine (*Pinus sylvestris* L.) and spruce (*Picea abies* (L.) H.Karst) industrial fibers (IKEA Industry Poland Sp. z o. o. brand Orla, Szczecin, Poland). The fibers were dried to a moisture content (MC) of about 4%. The MDF boards were bonded with urea–formaldehyde resin (UF; Silekol S123, Silekol Sp. z o.o., Kędzierzyn Koźle, Poland), with a formaldehyde to urea (F:U) molar ratio of 0.89, pH of 9.6, viscosity of 470 mPa s, and resination of 12%, the latter of which is commonly used in industry. The PLUSICE Organic Range A22 powder PCM used in the study was provided by Phase Change Material Products Ltd. (Unit 32, Mere View Industrial Estate, Yaxley, Cambridgeshire, PE7 3HS, UK). The basic characteristics of the PCM are shown in Table 1. 

### 2.2. Preparation of Panels

The test material consisted of laboratory-produced dry-formed fiberboards, each with a target density of 750 kg m^−3^, dimensions of 320 mm × 320 mm, and a nominal thickness of 16 mm. Each panel type was produced in triplicate. The panels were made in several variants: reference panels and panels containing various proportions (*w/w*) of PCM particles (5%, 10%, 30%, and 50% by weight relative to the board weight; Table 2), added during the resination process. Reference panels were manufactured without any PCM particles. The wood fibers were separated into three layers before resination: one inner layer (68% by weight) and two outer layers (each 16%). The PCM particles were incorporated only into the inner layer fibers during the resination process. The mats were manually formed. The boards were pressed using a hydraulic press (AKE, Mariannelund, Sweden) under the following conditions: a temperature of 180 °C, a pressing factor of 20 s/mm of nominal thickness, and a maximum unit pressing pressure of 2.5 MPa. After production, the boards were stored at 20 °C and 65% humidity until they reached a constant weight.

### 2.3. Characterization of the MDF Panels

The properties of the panels were determined in accordance with European Standards: density [45], modulus of rupture (MOR) and modulus of elasticity (MOE) [46], internal bond (IB) [47], screw withdrawal resistance (SWR) [48], water absorption (WA), and thickness swelling (TS) after 2 and 24 h of immersion [49]. No less than 12 samples per panel type were used to complete the mentioned tests. To evaluate the density profiles, the samples were cut into 50 mm × 50 mm test specimens (3 samples per panel type; after initial evaluation of results, one representative profile was used for final evaluation). The density profile was measured on a Grecon DA-X measuring instrument (Alfeld, Germany) with direct scanning X-ray densitometry across the panel thickness with an incremental step of 0.02 mm. All the mechanical tests were performed on a computer-controlled universal testing machine (Research and Development Centre for Wood-Based Panels Sp. z o.o., Czarna Woda, Poland). The thermal properties of the tested panel were investigated on 2 samples per variant, with dimensions of 300 mm × 300 mm, on the measurement unit designed for this research, consisting of a heating plate with a constant temperature of 40 °C. The sample with a temperature of 20 °C was placed on the plate, and the thermocouple connected to the computer was fitted to the sample surface. The sample surface temperature was registered every 60 s to reach the temperature of 40 °C ± 1 °C. The thermal conductivity coefficient was measured using the transient plane source (TPS) method with a TPS 2500S apparatus (Hot Disk AB, Göteborg, Sweden), featuring a sensor radius of 6.403 mm. The power was set to 10 mW, and measurements were conducted over a duration of 160 s at a temperature of 22 °C. Each measurement involved two samples with a thermal sensor placed between them. This test measures thermal conductivity, defined as the amount of heat conducted through a unit thickness of the material per unit time and area. For each sample, at least three test specimens were measured to ensure accuracy. All achieved results, whenever possible, were referred to the requirements of the proper European Standards [50].

### 2.4. Statistical Analysis

Using the RStudio software (2024.04.2 Build 764 © 2009–2024 Posit Software, PBC R Foundation, Vienna, Austria), statistical analysis was performed based on ANOVA tests, one-way for MOR, MOE, SWR, and IB, and two-way for thermal properties, TS, and WA. After showing statistically significant differences by the ANOVA test, Tukey’s post-hoc test was performed, resulting in homogeneous groups, which translated into an analysis of the significance of differences between variants.

## 3. Results and Discussion

### 3.1. Determination of Modulus of Rupture and Modulus of Elasticity in Bending

**Figure 1:**
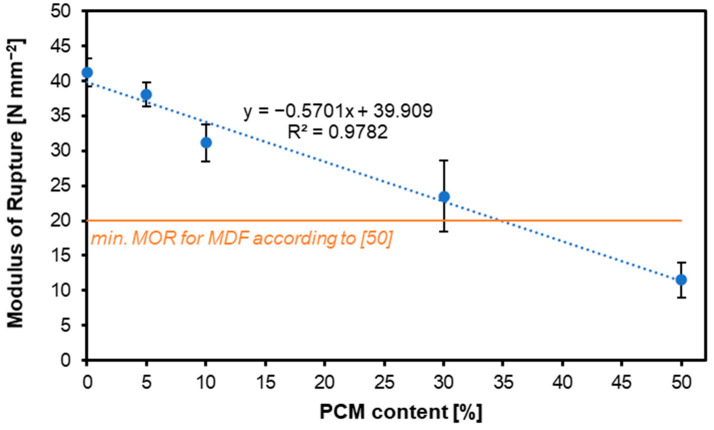
Influence of various contents of PCM on the MOR of produced MDF.

Figure 1 presents the results of the MOR tests of the panels considered. The trend line shown in the figure shows that as the PCM share increased, the MOR values decreased. For the variants containing PCM: 0%, 5%, 10%, 30%, and 50%, the MOR was: 41.21 N mm^−2^, 38.02 N mm^−2^, 31.15 N mm^−2^, 23.52 N mm^−2^, and 11.49 N mm^−2^, respectively. The variant with a PCM content of 50% did not meet the requirements of European Standards for MDF boards for internal use.

**Figure 2:**
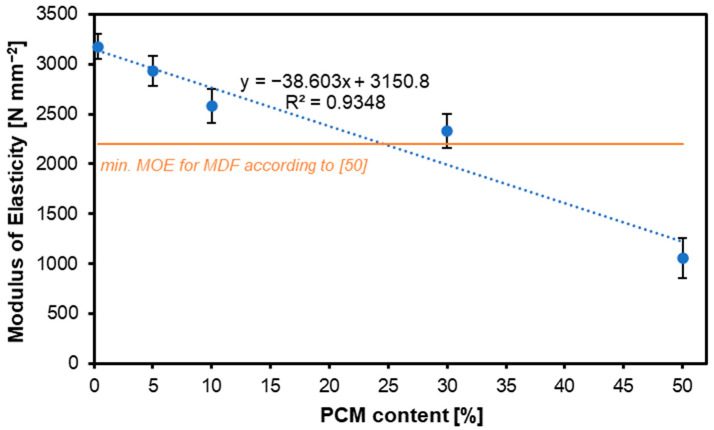
Influence of various contents of PCM on the MOE of produced MDF.

Figure 2 illustrates the results of the MOE examination of the considered panels. The tendency indicated by the trend line corresponds to the tendency for the MOR—here, too, increasing the share of PCM translates into a decrease in the obtained values. For the variants containing PCM: 0%, 5%, 10%, 30%, and 50%, the MOR was: 3176 N mm^−2^, 2932 N mm^−2^, 2579 N mm^−2^, 2331 N mm^−2^, and 1057 N mm^−2^, respectively. In the case of the MOE, the variant with a PCM content of 50% did not meet the requirements of European Standards for MDF boards for internal use, again.

The statistical analysis of the obtained MOR results for the tested boards showed no significant deviations between the variants containing 0% and 5% PCM, between the variants with 5% and 10% PCM content, and between the variants with 10% and 30% PCM content. The variant with a PCM of 50% differed statistically significantly from the other tested variants. The statistical analysis of the obtained MOE results for the tested boards showed a significant impact of the PCM share on the obtained values. No statistically significant differences were found between variants with a PCM content of 0%, 5%, and 10%, and no statistically significant deviations were found between the 5%, 10%, and 30% variants. The variant with a PCM content of 50% was characterized by MOE values that were statistically significantly different from the other variants.

Two types of PCMs were used in the study by [51]—microcapsules and liquid, with the examined MDF panels having a density above 700 kg m^−3^. All treatments had mean MOE values below the MOE of their corresponding control panel. Panels containing microencapsulated PCM showed lower MOE and MOR values than those made of liquid PCM. However, the trend indicates that an increase in the content of both microencapsulated and liquid PCM leads to an increase in MOE when bending the panel, which did not occur in our study with higher levels of PCM added. A panel with 6% liquid PCM and UF resin had the highest MOE of the panels developed, at 2072 N mm^−2^, followed by a panel using the same resin and PCM type, but at 2%, in 1974 N mm^−2^. On the other hand, the panel with 2% PCM microcapsules with PF resin exhibited the lowest value, at 1398 N mm^−2^. Like the MOE trend, all the treatments had MOR values lower than their respective control sample. The results obtained in our study for panels containing up to 30% of PCM showed values above 2000 N mm^−2^. 

### 3.2. Water Absorption and Thickness Swelling

**Figure 3:**
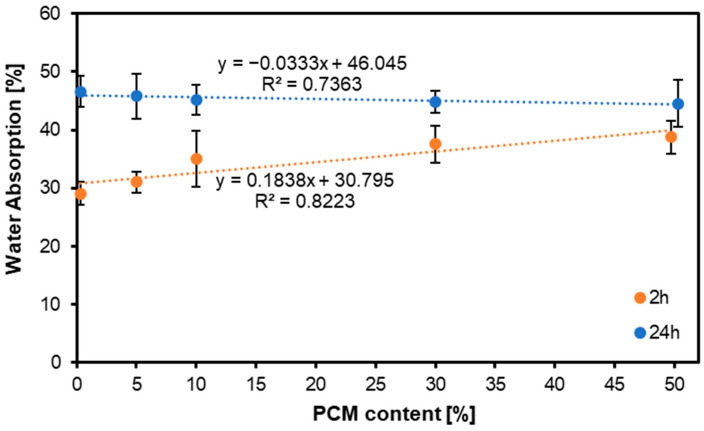
Water absorption of the MDF produced with the use of various contents of PCM.

Figure 3 illustrates the water absorption of MDF panels with varying PCM content. Over 2 h, a slight increase in water absorption was observed with increasing PCM content. However, after 24 h, it was noted that the dimensional stability of the MDF panels was not adversely affected by the addition of PCM particles. This study demonstrates that PCM particles do not exhibit significant water absorption capacity, thereby allowing the dimensional stability of the MDF panels to be maintained. Research on MDF boards with PCM additives was carried out by scientists in Chile, but a significant difference was the raw material, as old MDF boards were used to make the new ones, while the PCM additive in the highest concentration was 6%. However, these studies also confirmed what we observed in water absorption, that the addition of PCM did not have a significant effect on the dimensional stability of the MDF panels [51]; however, in recycled panels, the glue content of the old raw material often also reduces water absorption [52,53]. In addition, it is worth noting that the type of glue added also influenced the WA, and this was lower for phenol–formaldehyde glue than for urea–formaldehyde glue [51].

Statistical analysis showed that after 2 h of soaking in water, the WA values did not differ statistically significantly for the variants with 0% and 5% PCM. The same relationship occurred for variants containing 10% and 30% PCM, as well as between variants containing 30% and 50% PCM. However, after 24 h of soaking, statistical analysis showed no statistically significant differences between the values obtained for the tested variants.

**Figure 4:**
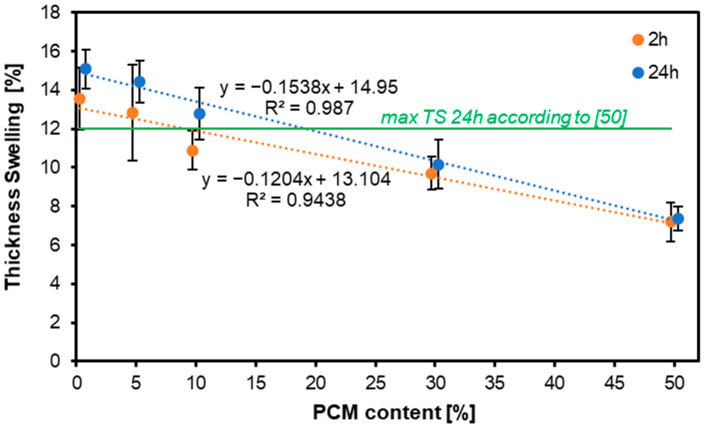
Thickness swelling of the MDF produced with the use of various contents of PCM.

Figure 4 shows the thickness swelling (TS) of MDF panels with the addition of PCM. After both 2 and 24 h, the thickness swelling for the different variants was smaller the higher the PCM content, the lower the TS. This clearly demonstrates the inability of PCM to absorb water. In practice, this means that the lower the amount of material capable of absorbing water, the lower the TS, as seen in the presented samples. This is expected to be due to the hydrophobic nature of the polymeric PCM coating, which does not absorb water. As the paraffin core is encapsulated in the coating, differences in its composition will not affect water absorption [54]. Additionally, the ratio of surface layer density to core density in MDF has a substantial negative association with the thickness swelling rate, implying that density affects swelling [55].

Statistical analysis showed that there were no statistically significant differences in the results obtained for TS between the variants containing 0% and 5% PCM, both after 2 h and after 24 h of soaking in water. The results obtained after 2 h of soaking showed that the variants with 10% and 30% PCM did not differ significantly from each other, and the same relationship was shown for the 30% and 50% variants. However, measurements collected after 24 h of soaking in water showed a variable relationship; variants with a PCM content of 10%, 30%, and 50% differed statistically significantly.

### 3.3. Thermal Properties

**Figure 5:**
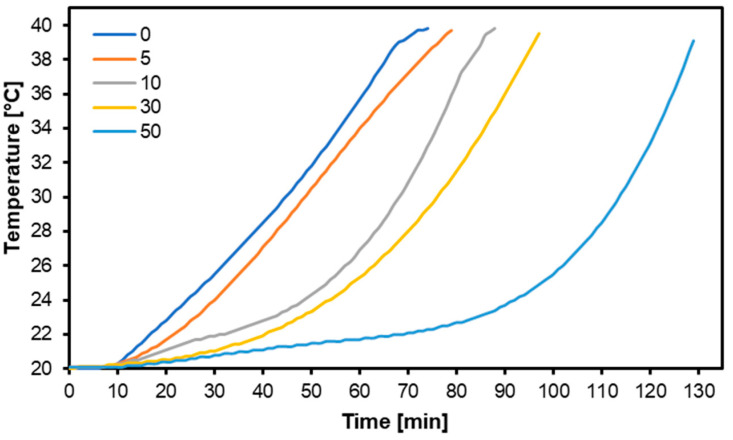
Thermal properties of MDF produced with different contents of PCM.

The thermal properties of MDF produced with different contents of PCM are presented in Figure 5. The best thermal properties were obtained for the variant where PCM constituted 50 parts by mass. The general trend was as follows: the higher the PCM content in the panel, the better the thermal properties. Statistical analysis showed that the thermal properties showed no statistically significant differences between the behavior of the variants with 0% and 5% PCM, and there were no significant deviations between the thermal behavior of the variants containing 10% and 30% PCM, as well as between the variants with 30% and 50% PCM. In Table 3, the results of the thermal conductivity coefficient have been collected. As can be seen, the thermal conductivity coefficient slightly increases with the PCM content increase. The reason is that the thermal conductivity coefficient of PCM used in the research (0.18 W mK^−1^; Table 1) is higher than the thermal conductivity of the MDF panels, that is, about 0.14 W mK^−1^ [56].

Similar results regarding the impact of PCM on MDF were observed in studies where fibers from recycled MDF boards were impregnated with liquid PCM [57]. The research was conducted on a decorative composite made of MDF inside which a pouch containing PCM was placed and then sealed with an HDF panel. Improved thermal properties were observed in the area where these decorative panels were applied. However, it was noted that the MDF and HDF panels themselves may hinder the operation of the PCM enclosed inside [58]. Another example of PCM application in wood composite materials involves embedding PVC tubes into fiberboard panels and subsequently filling them with PCM. This process is quite complex and requires precision. Additionally, such panels may serve more as decorative rather than structural panels. However, in terms of thermal properties, improved thermal conductivity has also been noted in this case [59]. Researchers found that the origin of PCMs impacts the thermal properties of the produced composites, particularly concerning temperature stability; for example, the type of PCM used, whether inorganic or organic, affects the thermal behavior of the composites [60].

### 3.4. Screw Withdrawal Resistance

**Figure 6:**
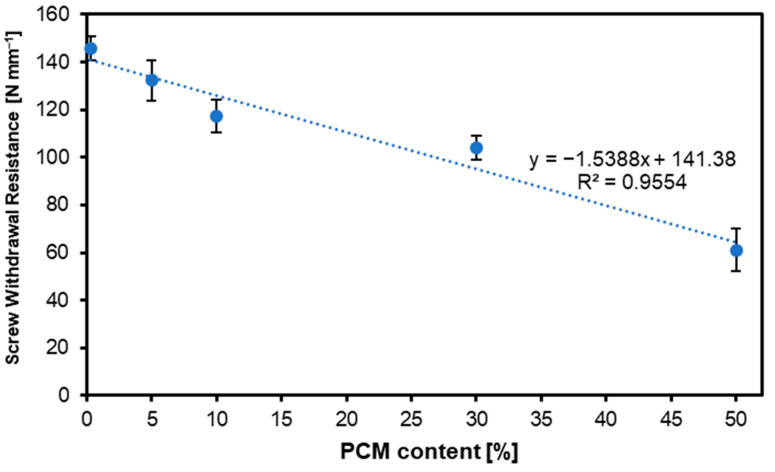
Screw withdrawal resistance of the MDF produced with the use of various contents of PCM.

Figure 6 shows the SWR test results of the considered panels. As in the case of the mechanical properties’ tests shown above, also in the case of SWR, the tendency of value changes with increasing PCM share is downward. For the variants with a PCM content of 0%, 5%, 10%, 30%, and 50%, respectively, the SWR values were: 146 N mm^−1^, 132 N mm^−1^, 117 N mm^−1^, 104 N mm^−1^, and 61 N mm^−1^. The SWR tests conducted on HDF panels with added recycled HDPE did not show significant differences between the HDPE content and the SWR of the tested samples [61]. However, it is worth mentioning that the plastic analyzed has a more plasticized structure. Due to the PCM structure, there may be a decrease in SWR parameters, given the lack of plasticity in the PCM structure in solid form, PCMs’ brittleness in the solid phase is controlled by their phase transition hysteresis, which is the difference between melting and solidification temperatures [62]. Adding fillers and plasticizers to PCM formulations can increase their relaxation qualities, lower internal tensions, and improve their strength and performance [63]. Statistical analysis of the obtained SWR results for the tested boards showed statistically significant differences between the results for all considered variants.

### 3.5. Internal Bond

**Figure 7:**
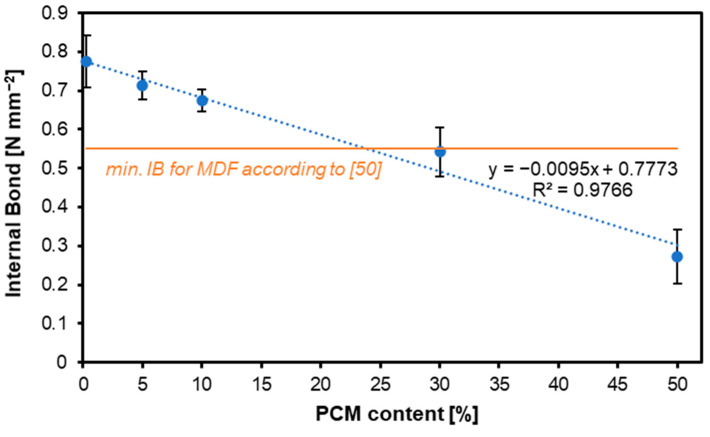
Internal bond of the MDF produced with the use of various contents of PCM.

Figure 7 shows the results of the IB test for the panels considered. As in the case of previous mechanical properties tests, also in the case of IB, an increase in the PCM share translates into a decrease in the obtained values. For the variants with a PCM content of 0%, 5%, 10%, 30%, and 50%, respectively, the IB values were: 0.78 N mm^−2^, 0.71 N mm^−2^, 0.68 N mm^−2^, 0.54 N mm^−2^, and 0.27 N mm^−2^. Variants containing PCM in the share of 30% and 50% did not meet the requirements set by the European norms for MDF for interior purposes. Statistical analysis of the IB values obtained for the tested boards showed no significant differences between the results obtained for the variants with a PCM content of 0%, 5%, and 10%. Also, no statistically significant deviations were observed between variants with a PCM content of 10% and 30%. The results for the variant with a PCM content of 50% differed statistically significantly from the other variants.

In the study by Rodríguez et. al. [51], the highest IB strength at 1.30 MPa was demonstrated by an MDF board with liquid PCM at 6% and UF resin, followed by a panel with the same type of PCM at 2% and UF resin at 1.21 MPa. The lowest IB strength was observed for the panel with microencapsulated PCM at 6% and PF resin at 0.83 MPa, which was 23.2% lower than its control panel (without PCM added). In some cases, modifications of MDF panels can lead to a weakening of certain properties, including internal bond (IB) strength. However, it is useful to know methods that can prevent such situations. One solution is the addition of crosslinking agents, such as polymeric-methylene diphenyl diisocyanate (pMDI) or glyoxal, which significantly increase the IB strength of MDF panels [64].

### 3.6. Density Profiles

**Figure 8:**
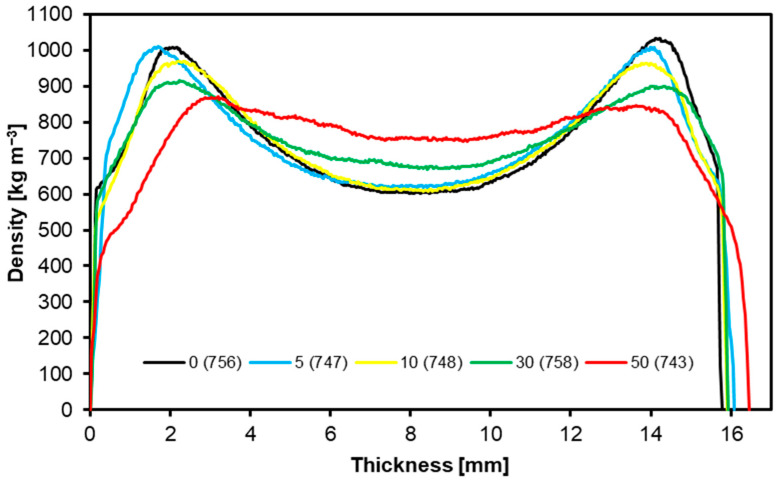
Density profiles of tested samples.

Figure 8 shows the density profiles of the tested panels. The average panel densities are 756 kg m^−3^, 747 kg m^−3^, 748 kg m^−3^, 758 kg m^−3^, and 743 kg m^−3^ for variants with a PCM content of 0%, 5%, 10%, 30%, and 50%. All considered variants showed U-shaped density profiles; however, in the case of the variant with a PCM share of 50%, the shape is flatter, and there is no visible density of layers at the panel surface. In the outer layers, the density of the boards was approximately 900 kg m^−3^ for the variants with 30% and 50% PCM, while for the remaining variants, the density at the board surfaces was close to 1000 kg m^−3^. In the center of the boards, their densities showed the lowest values, reaching approximately 600 kg m^−3^ for the variants with 0%, 5%, and 10% PCM, while for the variants with 30% and 50% PCM, these values were approximately 700 kg m^−3^ and 800 kg m^−3^, respectively. As confirmed by the study [65], the shape of a commercial MDF board resembles the density profiles acquired in this study, except for the variant containing 50% PCM, which deviates from the typical density profile of MDF. The density distribution through the thickness of MDF panels exhibits a notable correlation with the thickness swelling rate, internal bond strength, and static bending strength [55].

## 4. Conclusions

This study aimed to present the technology for producing thermally active MDF boards for interior applications. Based on the results obtained, it was shown that boards with the addition of powder PCM at the level of 50% do not meet the requirements set by European Standards for MDF boards for internal use regarding mechanical properties. It was noticed that an increase in the share of PCM in the plates translates into a decrease in the MOR, MOR, IB, and SWR values. In the case of WA, increased absorption was noticed with an increase in the PCM share; however, in the case of TS, only variants with 30% and 50% PCM content met the requirements of European Standards, while the remaining variants showed TS values that were too high. In the case of thermal properties, a positive effect of increasing the share of PCM in the boards was noticed, and the best results were obtained for the variant with a PCM share of 50%. Considering all the examined physical and mechanical properties, the ideal PCM content in MDF panels is 28%. In this case, MDF panels can successfully be used as material for furniture production, consistent with their original purpose. This study is innovative due to the combination of phase change materials with fiberboard for interior furnishing, using simple technology for producing boards for the furniture industry. This is a unique method, and with carefully chosen PCM amounts, it might be future proof. Such wood-based composites could perform as a good solution for furniture in offices and workspaces, providing better conditions by capturing and releasing heat, enabling more convenient usage of rooms. Therefore, it is worthwhile to continue developing the technology. The further attempts will be directed to optimize the panel’s properties to avoid “bottleneck” features, for example, in the case of mechanical properties. A crucial task to optimize may also be the technique and location of the phase change materials to the composites.

## Figures and Tables

**Table 1:** Properties of the PCM used in the research.

PCM—Properties	
Phase change temperature	22 °C
Density	785 kg m^−3^
Latent heat capacity	160 kJ kg^−1^
Volumetric heat capacity	126 MJ m^−3^
Specific heat capacity	2.2 kJ kgK^−1^
Thermal conductivity	0.18 W mK^−1^
Maximum operating temperature	200 °C

**Table 2:** The varieties of the tested panels.

Varieties of Tested Panels
Reference panels (without the addition of PCM)
Panels containing 5% of PCM particles
Panels containing 10% of PCM particles
Panels containing 30% of PCM particles
Panels containing 50% of PCM particles

**Table 3:** The thermal conductivity coefficient of the tested panels.

PCM Content (*w*/*w*)	Thermal Conductivity coef.	Std. dev.
[%]	[W mK^−1^]	
0	0.14	0.002
5	0.15	0.002
10	0.15	0.003
30	0.16	0.003
50	0.17	0.003

## Data Availability

https://doi.org/10.18150/MFSVKM.
